# Supplementary figures and images for: Comparative genomics of the proteostasis network in extreme acidophiles
Source: PLoS One. 2023 Sep 8;18(9):e0291164. doi: 10.1371/journal.pone.0291164 (PMC10490939; doi:10.1371/journal.pone.0291164)

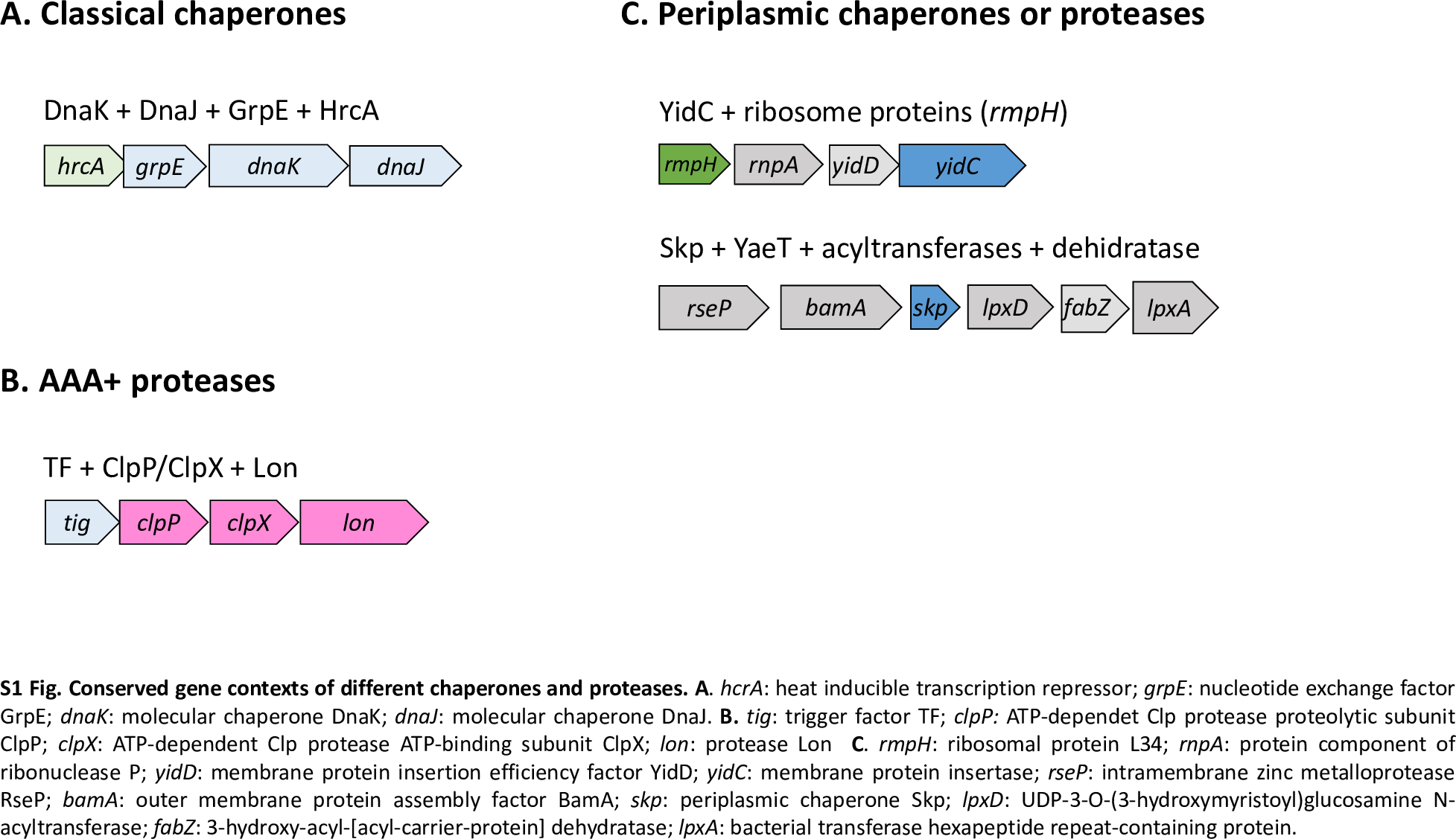

Supplement: S1 Fig — (TIF) [file pone.0291164.s001.tif]

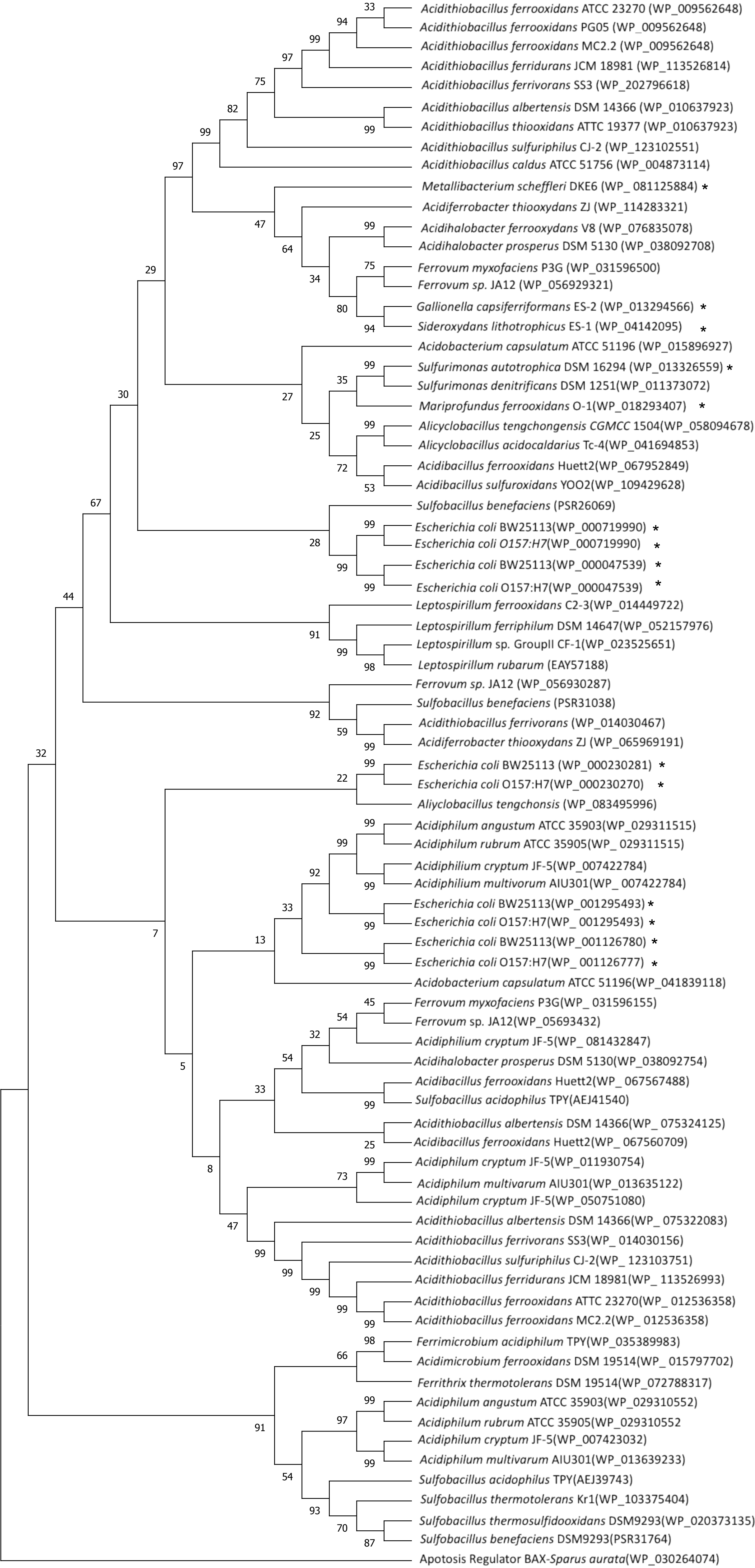

(\*) Neutrophiles

S2 Figure. Phylogenetic tree of RidA in acidophiles by maximum likelihood method

Supplement: S2 Fig — (PDF) [file pone.0291164.s002.pdf]

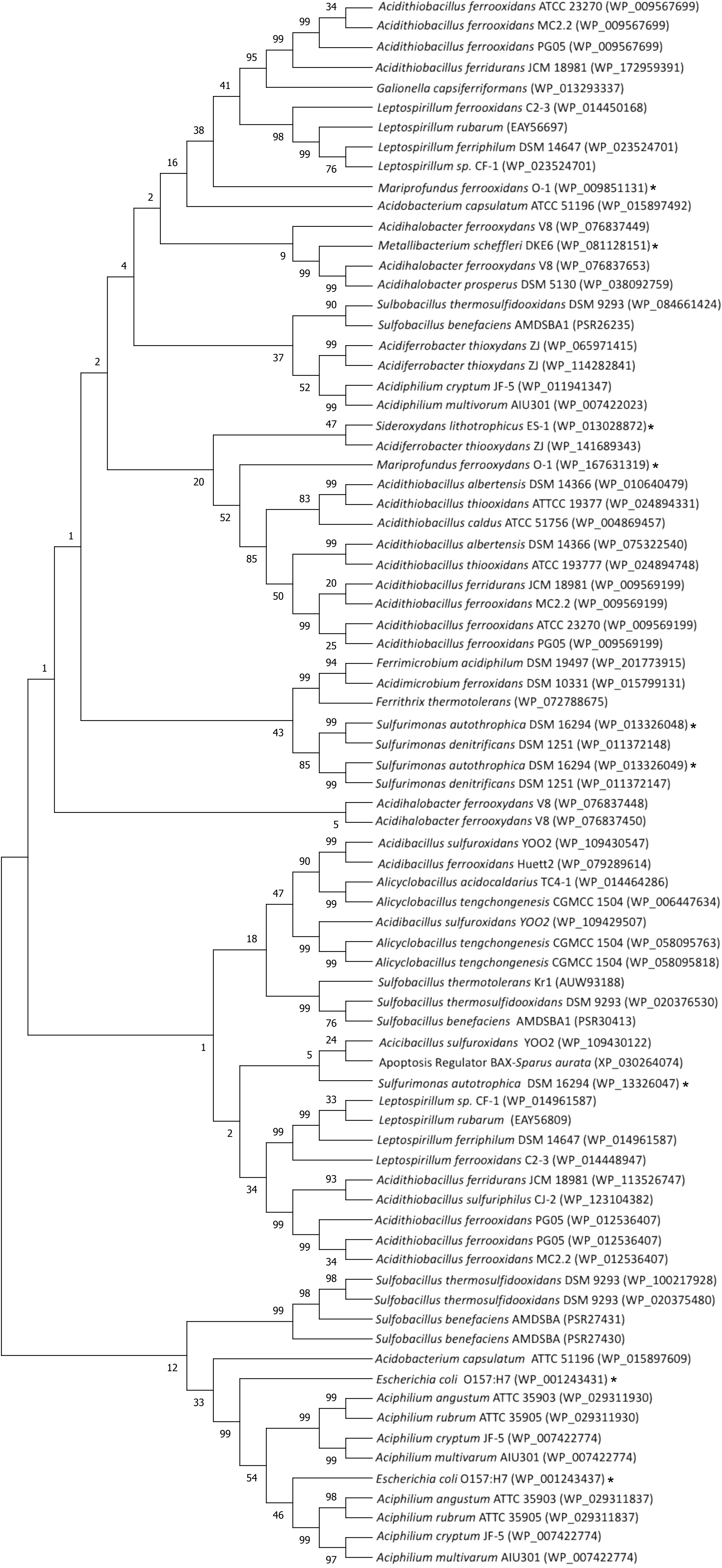

(\*) Neutrophiles

S3 Figure. Phylogenetic tree of Hsp20 in acidophiles by maximum likelihood method

Supplement: S3 Fig — (PDF) [file pone.0291164.s003.pdf]
