# Supplementary material for: Comparative genomics of the proteostasis network in extreme acidophiles
Source: PLoS One. 2023 Sep 8;18(9):e0291164. doi: 10.1371/journal.pone.0291164 (PMC10490939; doi:10.1371/journal.pone.0291164)
Supplement: Attachment — Submitted filename: Reviewer Attachments.docx [file pone.0291164.s005.docx]

It would be beneficial to begin the introduction with references to the influence of acidic pH, high concentrations of dissolved metals and high osmolarity on proteostasis Network in order to enhance the value of the study. This article can be included after some revisions.

1. The resolution of most Figures are too low to print for publisher.

2. Why did authors choose the 40 acidophilic bacteria mentioned in the manuscript as the research object, and what are the unique features of these bacteria.

3. The content is plentiful, but some part of the reference literatures is kind of obsolete (in 5 years). Key publications should be cited as completed as possible.
