# Supplementary material for: Comparative genomics of the proteostasis network in extreme acidophiles
Source: PLoS One. 2023 Sep 8;18(9):e0291164. doi: 10.1371/journal.pone.0291164 (PMC10490939; doi:10.1371/journal.pone.0291164)
Supplement: Attachment — Submitted filename: Answer to the Reviewers PONE-D-23-16802.docx [file pone.0291164.s006.docx]

**Answer to the Reviewers PONE-D-23-16802**

We thank the effort and work of the reviewers in order to improve the quality and clarity of our manuscript. We have followed the advices and below you can find answers to the point raised by the reviewers 1 and 2. It is of note that Figures 2 and 6 have been modified so that only a part of the phylogenetic tree is presented, while the full tree is attached in supporting information as figures S2 and S3.

**Reviewer #1**

**Many of the analyzed genomes do not belong even to moderately acidophilic microorganisms, while the authors state that these bacteria are extreme acidophiles. There are also incorrect statements about some autotrophic, mixotrophic, or heterotrophic bacteria.**

**Please check relevant information about all bacteria used in this analysis and revise the text throughout the manuscript, including l. 274–275 and Table S1. If this research is focused on extreme acidophiles, only bacterial genomes of acidophilic microorganisms should be included. Otherwise, the concept and focus of this research should be changed.**

**Answer.** We have carefully inspected the information related to all microorganisms analysed. Then, when corresponded we modified the categories as suggested by reviewer 1. The information related to metabolism and acidophilic/neutrophilic character of each bacterium was modified in the S1 Table and also in the text of the whole manuscript. One exception was *Acidimicrobium ferrooxidans* which oxidizes ferrous iron, but also fix CO2 and organic carbon as carbon sources, for that it was classified as mixotrophic (not heterotroph as suggested by the reviewer 1). Below, we have attached a list that indicates the character of each microorganism and contains the corresponding reference.

The revised version of the manuscript clearly indicates in the text the acidophilic or neutrophilic character of the bacteria. As indicated the main purpose is characterizing the proteostasis network in the acidophilic microorganisms; thus we have carried out the analysis using acidophilic bacteria genomes. However, the neutrophilic bacteria were also used for comparison purposes. The general conclusion that was initially stated was not affected or contradicted.

**Reviewer #2**:

**a. It would be beneficial to begin the introduction with references to the influence of acidic pH, high concentrations of dissolved metals and high osmolarity on proteostasis Network in order to enhance the value of the study. This article can be included after some revisions.**

**Answer.** The referred information by the reviewer 2 was in fact included in the third paragraph of the original manuscript. Now, we have slightly modified the text to include osmolarity as an influential factor on the proteostasis. However, we prefer to keep the original order of the **Introduction** section, where a general description about proteostasis concept was given first, and then a brief discussion about the effect of environmental factors. We, hope the reviewer can accept to keep this structure.

**b. The resolution of most Figures are too low to print for publisher.**

**Answer.** We have improved the quality of the figures. We hope it meet the requirements of the journal to this regard. In additional, the Figures 2 and 6 were have been modified so that only a part of the phylogenetic tree is presented, while the full tree is attached in supporting information as figures S2 and S3.

**c. Why did authors choose the 40 acidophilic bacteria mentioned in the manuscript as the research object, and what are the unique features of these bacteria**.

**Answer.** The number of total acidophilic bacteria described so far amounts to a few tens; so the number itself is quite small. In addition, we selected those bacteria whose complete genome were available. In general, the genomes available in the databases correspond to the best-studied bacteria (e.g. *Acidithiobacillus*), therefore they were included in our analysis.

**d. The content is plentiful, but some part of the reference literatures is kind of obsolete (in 5 years). Key publications should be cited as completed as possible.**

**Answer.** In order to accomplish the requirement, the complete set of references was revised and checked. Finally, we managed to replace 15 references by equivalent and more recent works.

**Metabolic characteristics of the bacteria used in this study** (this table is included for purposes of revision only; in the manuscript we have just modified the S1 Table).

|  | **Bacteria** | **Carbon metabolism** | **Optimal pH range** | **Doi** |
| --- | --- | --- | --- | --- |
| 1 | *Acidithiobacillus albertensis* DSM14366 | Autotrophic | Acidophilic | [10.1186/s40793-017-0282-y](https://doi.org/10.1186/s40793-017-0282-y) |
| 2 | *Acidithiobacillus caldus* ATCC51756 | Autotrophic | Acidophilic | [10.1128/JB.00843-09](http://dx.doi.org/10.1128/JB.00843-09) |
| 3 | *Acidithiobacillus ferridurans* JCM18981 | Autotrophic | Acidophilic | [10.1128/MRA.01028-18](http://dx.doi.org/10.1128/MRA.01028-18) |
| 4 | *Acidithiobacillus ferrivorans* SS3 | Autotrophic | Acidophilic | [10.1007/s00792-016-0882-2](https://link.springer.com/article/10.1007/s00792-016-0882-2) |
| 5 | *Acidithiobacillus ferrooxidans* ATCC23270 | Autotrophic | Acidophilic | [10.3389/fmicb.2011.00079](http://dx.doi.org/10.3389/fmicb.2011.00079) |
| 6 | *Acidithiobacillus thiooxidans* ATCC19377 | Autotrophic | Acidophilic | [10.1186/s40793-017-0305-8](http://dx.doi.org/10.1186/s40793-017-0305-8) |
| 7 | *Acidithiobacillus sulfuriphilus* CJ-2 | Autotrophic | Acidophilic | [10.1099/ijsem.0.003576](http://dx.doi.org/10.1099/ijsem.0.003576) |
| 8 | *Acidithiobacillus* sp. PG05 | Autotrophic | Acidophilic | [10.3389/fmicb.2022.960324](http://dx.doi.org/10.3389/fmicb.2022.960324) |
| 9 | *Acidithiobacillus ferrooxidans* MC 2.2 | Autotrophic | Acidophilic | [10.3389/fmicb.2022.960324](http://dx.doi.org/10.3389/fmicb.2022.960324) |
| 10 | *Acidiphilium angustum* ATCC 35903 | Heterotrophic | Acidophilic | [10.1099/00207713-36-2-197](https://doi.org/10.1099/00207713-36-2-197) |
| 11 | *Acidiphilium cryptum* JF-5 | Heterotrophic | Acidophilic | [10.1016/j.hydromet.2015.08.003](http://dx.doi.org/10.1016/j.hydromet.2015.08.003) |
| 12 | *Acidiphilium multivorum* AIU301 | Heterotrophic | Acidophilic | [10.3390/microorganisms9050984](https://doi.org/10.3390%2Fmicroorganisms9050984) |
| 13 | *Acidiphilium rubrum* ATCC 35905 | Heterotrophic | Acidophilic | 10.1099/00207713-36-2-197 |
| 14 | *Ferrovum myxofaciens* P3G | Autotrophic | Acidophilic | [10.1128/AEM.03230-13](http://dx.doi.org/10.1128/AEM.03230-13) |
| 15 | *Ferrovum* sp. JA12 | Autotrophic | Acidophilic | [10.1371/journal.pone.0146832](http://dx.doi.org/10.1371/journal.pone.0146832) |
| 16 | *Gallionella capsiferriformans* ES-2 | Autotrophic | Neutrophilic | [10.1111/gbi.12162](https://doi.org/10.1111/gbi.12162) |
| 17 | *Sideroxydans lithotrophicus* ES-1 | Autotrophic | Neutrophilic | [10.1093/femsec/fiz034](http://dx.doi.org/10.1093/femsec/fiz034) |
| 18 | *Acidiferrobacter thiooxydans* ZJ | Autotrophic | Acidophilic | [10.1016/j.resmic.2018.08.001](https://doi.org/10.1016/j.resmic.2018.08.001) |
| 19 | *Acidihalobacter ferrooxydans* V8 | Autotrophic | Acidophilic | [10.1128/genomeA.00413-17](http://dx.doi.org/10.1128/genomeA.00413-17) |
| 20 | *Acidihalobacter prosperus* DSM5130 | Autotrophic | Acidophilic | [10.1128/genomeA.01042-14](http://dx.doi.org/10.1128/genomeA.01042-14) |
| 21 | *Metallibacterium scheffleri* DKE6 | Mixotrophic | Neutrophilic, acid-tolerant | [10.1093/femsec/fix011](https://doi.org/10.1093/femsec/fix011) |
| 22 | *Escherichia coli* O157:H7 str. Sakai DNA | Heterotrophic | Neutrophilic | [10.1128/JB.01481-08](https://doi.org/10.1128%2FJB.01481-08) |
| 23 | *Escherichia coli* BW25113 | Heterotrophic | Neutrophilic | [10.1099/mic.0.000742](https://doi.org/10.1099/mic.0.000742) |
| 24 | *Mariprofundus ferroxydans* O-1 | Autotrophic | Neutrophilic | 10.1007/s12275-014-3625-z |
| 25 | *Sulfurimonas autotrophica* DSM16294 | Autotrophic | Neutrophilic | 10.1099/ijs.0.02682-0 |
| 26 | *Sulfurimonas denitrificans* DSM1251 | Autotrophic | Acidophilic | [10.3389/fmicb.2015.00989](https://doi.org/10.3389/fmicb.2015.00989) |
| 27 | *Leptospirillum ferrooxidans* C2-3 | Autotrophic | Acidophilic | 10.1128/jb.00696-12 |
| 28 | *Leptospirillum ferriphilum* DSM14647 | Autotrophic | Acidophilic | [10.1128/genomeA.01153-14](http://dx.doi.org/10.1128/genomeA.01153-14) |
| 29 | *Leptospirillum* sp. CF-1 | Autotrophic | Acidophilic | [10.1016/j.jbiotec.2016.02.008](https://doi.org/10.1016/j.jbiotec.2016.02.008) |
| 30 | *Leptospirillum rubarum* | Autotrophic | Acidophilic | [10.1128/AEM.02943-08](http://dx.doi.org/10.1128/AEM.02943-08) |
| 31 | *Acidibacillus sulfuroxidans* Y002 | Heterotrophic | Acidophilic | [10.1016/j.resmic.2022.104008](https://doi.org/10.1016/j.resmic.2022.104008) |
| 32 | *Acidibacillus ferrooxidans* Huett2 | Heterotrophic | Acidophilic | 10.4028/www.scientific.net/SSP.262.334 |
| 33 | *Alicyclobacillus acidocaldarius* subsp. *acidocaldarius* Tc-4-1 | Heterotrophic | Acidophilic | [10.1128/JB.05709-11](https://dx.doi.org/10.1128%2FJB.05709-11) |
| 34 | *Alicyclobacillus tengchongensis* CGMCC1504 | Heterotrophic | Acidophilic | [10.1007/s12275-014-3625-z](http://dx.doi.org/10.1007/s12275-014-3625-z) |
| 35 | *Sulfobacillus acidophilus* TPY | Mixotrophic | Acidophilic | [10.3389/fmicb.2016.01861](http://dx.doi.org/10.3389/fmicb.2016.01861) |
| 36 | *Sulfobacillus thermosulfidooxidans* DSM9293 | Mixotrophic | Acidophilic | [10.3389/fmicb.2020.00044](https://doi.org/10.3389/fmicb.2020.00044) |
| 37 | *Sulfobacillus thermotolerans* Kr1 | Mixotrophic | Acidophilic | [10.1038/s41598-019-51486-1](https://www.nature.com/articles/s41598-019-51486-1) |
| 38 | *Sulfobacillus benefaciens* | Mixotrophic | Acidophilic | [10.1007/s00792-008-0184-4](http://dx.doi.org/10.1007/s00792-008-0184-4) |
| 39 | *Acidobacterium capsulatum* ATCC 51196 | Mixotrophic | Acidophilic | 10.1128/AEM.02294-08 |
| 40 | *Ferrimicrobium acidiphilum* DSM 19497 | Heterotrophic | Acidophilic | [10.1099/ijs.0.65409-0](http://dx.doi.org/10.1099/ijs.0.65409-0) |
| 41 | *Ferrithrix thermotolerans* DSM 19514 | Heterotrophic | Acidophilic | [10.1099/ijs.0.65409-0](http://dx.doi.org/10.1099/ijs.0.65409-0) |
| 42 | *Acidimicrobium ferrooxidans* DSM 10331 | Mixotrophic | Acidophilic | 10.4056/sigs.1463 |
